# Association of Physical Activity and Fracture Risk Among Postmenopausal Women

**DOI:** 10.1001/jamanetworkopen.2019.14084

**Published:** 2019-10-25

**Authors:** Michael J. LaMonte, Jean Wactawski-Wende, Joseph C. Larson, Xiaodan Mai, John A. Robbins, Meryl S. LeBoff, Zhao Chen, Rebecca D. Jackson, Andrea Z. LaCroix, Judith K. Ockene, Kathleen M. Hovey, Jane A. Cauley

**Affiliations:** 1 Department of Epidemiology and Environmental Health, University at Buffalo, The State University of New York, Buffalo; 2 Data Coordinating Center, Fred Hutchinson Cancer Research Center, Seattle, Washington; 3 Department of Medicine, University of California, Davis, Sacramento; 4 Division of Endocrine, Diabetes, and Hypertension, Brigham and Woman’s Hospital, Harvard Medical School, Boston, Massachusetts; 5 Division of Epidemiology and Biostatistics, Mel and Enid Zuckerman College of Public Health, University of Arizona, Tucson; 6 Division of Endocrinology, Diabetes and Metabolism, Department of Internal Medicine, The Ohio State University, Columbus; 7 Division of Epidemiology, Department of Family Medicine and Public Health, University of California, San Diego; 8 Division of Preventive and Behavioral Medicine, University of Massachusetts Medical School, Worcester; 9 Department of Epidemiology, Graduate School of Public Health, University of Pittsburgh, Pittsburgh, Pennsylvania

## Abstract

**Importance:**

Physical activity is inversely associated with hip fracture risk in older women. However, the association of physical activity with fracture at other sites and the role of sedentary behavior remain unclear.

**Objective:**

To assess the associations of physical activity and sedentary behavior with fracture incidence among postmenopausal women.

**Design, Setting, and Participants:**

The Women’s Health Initiative prospective cohort study enrolled 77 206 postmenopausal women aged 50 to 79 years between October 1993 and December 1998 at 40 US clinical centers. Participants were observed for outcomes through September 2015, with data analysis conducted from June 2017 to August 2019.

**Exposures:**

Self-reported physical activity and sedentary time.

**Main Outcomes and Measures:**

Hazard ratios (HRs) and 95% CIs for total and site-specific fracture incidence.

**Results:**

During a mean (SD) follow-up period of 14.0 (5.2) years among 77 206 women (mean [SD] age, 63.4 [7.3] years; 66 072 [85.6%] white), 25 516 (33.1%) reported a first incident fracture. Total physical activity was inversely associated with the multivariable-adjusted risk of hip fracture (>17.7 metabolic equivalent [MET] h/wk vs none: HR, 0.82; 95% CI, 0.72-0.95; *P *for trend < .001). Inverse associations with hip fracture were also observed for walking (>7.5 MET h/wk vs none: HR, 0.88; 95% CI, 0.78-0.98; *P *for trend = .01), mild activity (HR, 0.82; 95% CI, 0.73-0.93; *P *for trend = .003), moderate to vigorous activity (HR, 0.88; 95% CI, 0.81-0.96; *P *for trend = .002), and yard work (HR, 0.90; 95% CI, 0.82-0.99; *P *for trend = .04). Total activity was positively associated with knee fracture (>17.7 MET h/wk vs none: HR, 1.26; 95% CI, 1.05-1.50; *P *for trend = .08). Mild activity was associated with lower risks of clinical vertebral fracture (HR, 0.87; 95% CI, 0.78-0.96; *P *for trend = .006) and total fractures (HR, 0.91; 95% CI, 0.87-0.94; *P *for trend < .001). Moderate to vigorous activity was positively associated with wrist or forearm fracture (HR, 1.09; 95% CI, 1.03-1.15; *P *for trend = .004). After controlling for covariates and total physical activity, sedentary time was positively associated with total fracture risk (>9.5 h/d vs <6.5 h/d: HR, 1.04; 95% CI, 1.01-1.07; *P *for trend = .01). When analyzed jointly, higher total activity mitigated some of the total fracture risk associated with sedentary behavior. Analysis of time-varying exposures resulted in somewhat stronger associations for total physical activity, whereas those for sedentary time were materially unchanged.

**Conclusions and Relevance:**

In older ambulatory women, higher total physical activity was associated with lower total and hip fracture risk but higher knee fracture risk. Mild activity and walking were associated with lower hip fracture risk, a finding with important public health implications because these activities are common in older adults. The positive association between sedentary time and total fracture risk requires further investigation.

## Key Points

**Question:**

Is the amount and intensity of physical activity associated with total and site-specific fracture among postmenopausal women who participated in the Women’s Health Initiative study?

**Findings:**

In this cohort study of 77 206 postmenopausal women with a mean follow-up of 14 years, higher amounts of total, mild, moderate to vigorous, and walking physical activity were significantly associated with lower risk of hip fracture. Positive associations existed for moderate to vigorous physical activity with wrist or forearm fractures and for sedentary behavior with total fractures.

**Meaning:**

Regular physical activity, including lighter-intensity activities, and less sedentary time is associated with reduced risk of fracture in older women.

## Teaser

This cohort study of the Women’s Health Initiative (WHI) assesses the associations of physical activity and sedentary behavior with fracture incidence among postmenopausal women.

## Introduction

Approximately 1.5 million fractures occur annually in women who live in the United States, accounting for $12.7 billion in health care costs.^1^ Approximately 14% of fractures occur in the hip^1^; mortality after hip fracture is as high as 20%.^2^ Fracture has been associated with low bone mineral density (BMD), propensity to fall, and declines in muscle strength, balance, mobility, and physical functioning.^3,4,5^

The 2008 Physical Activity Guidelines Advisory Committee evaluated quality and quantity of evidence from 21 studies and concluded that people with higher total physical activity (PA) levels have 36% to 68% lower risk of hip fracture.^6^ The Advisory Committee Report for the 2018 revision of the PA guidelines did not include an explicit update on fracture outcomes but did indicate that evidence supports the conclusion that higher amounts of total PA are associated with lower risk of falls and fall-related injuries, including bone fracture.^7^ In both the 2008 and 2018 PA guidelines, consensus was lacking regarding fracture risk at sites beside the hip. The majority of published studies assessed PA as a composite measure; thus, the role of PA types and intensities in fracture is unclear. Sedentary behavior (eg, sitting time) is becoming an established modifiable risk factor for major forms of morbidity and mortality, independent of PA habits^7^; however, its contribution to fracture has not been systematically evaluated.^6,7^

The Women’s Health Initiative (WHI) is a prospective cohort study among postmenopausal women with ongoing assessment of fractures. We examined recreational PA, household activities, walking, and sedentary behavior in association with incident fracture and the extent to which age, race/ethnicity, or fall frequency modified this association in older, community-dwelling, ambulatory women.

## Methods

### Study Population

The WHI observational study design has been published.^8^ Recruitment of participants was conducted at 40 US clinic centers from October 1993 through December 1998, enrolling 93 676 postmenopausal women aged 50 to 79 years.^9^ Women with predicted survival of less than 3 years or with conditions that might compromise retention were ineligible. Study protocols were approved by institutional review boards at participating institutions. Written informed consent was obtained from participants. The initial WHI observational study concluded in 2005. Additional follow-up was obtained from women who consented to participate in 2 WHI Extension Studies (2005-2010 and 2010-2015). This study conformed to the Strengthening the Reporting of Observational Studies in Epidemiology (STROBE) reporting guideline.

### Assessment of PA and Sedentary Behavior

Participants completed baseline self-administered questionnaires asking frequency (days per week) and duration (minutes) of usual mild, moderate, and strenuous recreational PA.^10^ *Strenuous PA* was defined as exercise resulting in sweating and a fast heartbeat, such as aerobics, aerobics dancing, jogging, tennis, or swimming laps; *moderate PA*, less exhausting activities, such as biking outdoors, using an exercise machine, calisthenics, easy swimming, or popular or folk dancing; and *mild PA*, slow dancing, bowling, or golf. Walking was assessed separately from these activities with the following questions: “how often do you walk outside the home for more than 10 minutes without stopping,” “when you walk outside the home for more than 10 minutes without stopping, for how many minutes do you usually walk,” and “what is your usual speed.” Nonrecreational activities, including the time (hours per week) spent on heavy indoor household chores (ie, scrubbing floors, sweeping, or vacuuming) and yard work (ie, mowing, raking, gardening, or shoveling snow) were queried using questions specific to these constructs.

Physical activity was summarized as energy expenditure, calculated as the product of metabolic equivalent (MET) intensity values for each activity multiplied by the hours per week of reported participation (MET hours per week). Standard MET values were assigned to mild (3.0 METs), moderate (4.5 METs), and strenuous (7.0 METs) activity, 4 walking speeds (ie, slow, <2 mph, 2.0 METs; average or normal, approximately 2-3 mph, 3.0 METs; fairly fast, approximately 3-4 mph, 4.0 METs; and fast, >4 mph, 5.0 METs), heavy chores (3.5 METs), and yard work (4.0 METs).^11^

Sedentary behavior was assessed by self-reported usual time spent sitting (hours per day and night) including at work, at the table eating, driving or riding in a car or bus, watching television, or talking; and usual time spent lying down (hours per day and night), and resting but not asleep or watching television.^12^ Self-reported intensity-specific PA, walking, total PA, yard work, chores, and inactivity assessed by WHI questionnaires have demonstrated reproducibility (intraclass correlation coefficients, 0.51-0.77)^10,13^ and validity (Spearman ρ, 0.45-0.52 with accelerometer).^14^

### Ascertainment of Incident Fracture

Participants were observed from enrollment through September 2015 using annual mailed health questionnaires. As hip fracture was a primary outcome in the WHI, all hip fractures were adjudicated in the main study (1994-2005) and Extension Study I (2005-2010). Trained physicians reviewed radiology reports, with hospital discharge summaries, operative reports, and clinic and progress notes as additional sources. Self-reported fractures at sites other than the hip were not adjudicated. From 2010 to 2015, self-reported fractures at all sites including hip were not adjudicated. Self-reported fracture in WHI has good agreement (a mean of 76%) with criterion medical records.^15^ In the present study, we evaluated total PA in association with total and site-specific fracture end points. We then conducted additional analysis on specific PA types in association with a reduced set of fracture end points (ie, hip, wrist or forearm, and clinical vertebral), which are designated major osteoporotic fracture events in the WHI.^16^ This approach was used to reduce the total number of statistical comparisons involving secondary exposures and in subgroups where power may be limited for site-specific end points.

### Assessment of Other Variables

At enrollment, participants completed questionnaires regarding sociodemographic characteristics including self-reporting of race/ethnicity, family and personal medical history, medication and supplement use, and lifestyle factors. Physical function was assessed using the RAND-36.^10^ Weight and height were measured using a calibrated clinical scale and stadiometer.

### Statistical Analysis

Participant characteristics were assessed across total PA categories using analysis of variance and χ^2^ tests. Cox proportional hazards regression was used to estimate hazard ratios (HRs) and 95% CIs for associations with incident fracture. The primary exposure variable was total PA categorized as none (0 MET h/wk; referent group) and the following tertiles: more than 0 to 7.5 MET h/wk, more than 7.5 to 17.7 MET h/wk, and more than 17.7 MET h/week. Additional activity exposures were defined as follows: (1) yard work and chores, categorized as none and median of active participants; (2) walking, categorized as none and tertiles of more than 0 to 3.5 MET h/wk, more than 3.5 to 7.5 MET h/wk, and more than 7.5 MET h/wk; (3) mild activity, categorized as none, more than 0 to 3.5 MET h/wk, and more than 3.5 MET h/wk; and (4) moderate to vigorous activity, categorized as less than 9 MET h/wk and at least 9 MET h/wk, to be comparable with guideline-recommended levels. Time to first fracture was analyzed, with censoring at death, loss to follow-up, or the end of follow-up on September 30, 2015. Death was not an event in the models. Owing to differences in hip fracture ascertainment between WHI Extension Study II (ie, self-reported) and the rest of the study (ie, adjudicated), models evaluating hip and total fracture outcomes included a time-dependent stratification by hip ascertainment type within the Cox models.

Multivariable-adjusted models included the following covariates: age, race/ethnicity, education, smoking status, alcohol use, height, weight, history of fracture after age 55 years, bone drug use, corticosteroid use, calcium intake, vitamin D intake, lifetime hormone therapy use, falls in the past year, physical function, thiazide use, diabetes, age at menopause, and osteoporosis history. Additional models controlled mutually for sedentary time or PA to assess their independent association with fracture risk. The joint association of total PA and sedentary time with risk of total fracture was also examined. Multiplicative interactions for total PA with enrollment age, race/ethnicity, and fall frequency were explored using cross-product terms. Sensitivity analysis was conducted limiting follow-up to the first 10 years. Robustness of results from primary analyses using baseline PA and sedentary exposures were evaluated using time-varying exposure analysis based on updated information collected 3 and 6 years after baseline. *P* values were for 2-sided hypothesis tests conducted at a statistical significance of .05. Analyses were conducted from June 2017 to August 2019 using SAS version 9.4 (SAS Institute).

## Results

After exclusions (n = 3165) and missing covariates (n = 13 305) (eTable 8 in the Supplement), 77 206 women were included in this study. Baseline characteristics of the cohort appear in Table 1. Participants had a mean (SD) age of 63.4 (7.3) years at enrollment, were mainly white (66 072 [85.6%]), with more than a high school education (61 688 [79.9%]) and a low prevalence of current smoking (4663 [6.0%]), osteoporosis (6485 [8.4%]), and bone drug use (2986 [3.9%]). Slightly less than one-third (24 813 [32.1%]) reported at least 1 fall in the past year. Because of the large cohort size, most baseline characteristics were significantly associated with total PA, although, in some instances, absolute differences were modest (Table 1).

**Table 1.** Baseline Characteristics by Categories of Total Recreational Physical Activity

Characteristic	No. (%)					*P* Value^a^
	All Participants (N = 77 206)	Total Physical Activity, MET h/wk				
		0 (n = 10 127)	>0 to 7.5 (n = 22 904)	>7.5 to 17.7 (n = 21 705)	>17.7 (n = 22 470)	
Age, y						
Mean (SD)	63.4 (7.3)	63.1 (7.5)	63.6 (7.4)	63.5 (7.3)	63.2 (7.2)	<.001
No. (%)						
50-59	25 309 (32.8)	3531 (34.9)	7376 (32.2)	6949 (32.0)	7453 (33.2)	<.001
60-69	34 120 (44.2)	4278 (42.3)	9915 (43.3)	9717 (44.7)	10 210 (45.4)	
70-79	17 777 (23.0)	2318 (22.9)	5613 (24.5)	5039 (23.2)	4807 (21.4)	
Race/ethnicity						
White	66 072 (85.6)	8141 (8.4)	19 169 (83.6)	18 916 (87.1)	19 846 (88.3)	<.001
Black	5214 (6.8)	1068 (1.6)	1810 (7.9)	1271 (5.9)	1065 (4.7)	
Hispanic	2389 (3.1)	445 (4.4)	830 (3.6)	568 (2.6)	546 (2.4)	
American Indian	287 (0.4)	42 (0.4)	101 (0.4)	66 (0.3)	78 (0.3)	
Asian/Pacific Islander	2233 (2.9)	288 (2.8)	683 (3.0)	612 (2.8)	650 (2.9)	
Unknown	1011 (1.3)	143 (1.4)	311 (1.4)	272 (1.3)	285 (1.3)	
Education						
≤High school	15 518 (20.1)	3048 (3.1)	5497 (24.0)	3798 (17.5)	3191 (14.2)	<.001
>High school	28 103 (36.4)	3950 (39.0)	8726 (38.1)	7749 (35.7)	7685 (34.2)	
≥College degree	33 585 (43.5)	3129 (3.9)	8681 (37.9)	10 158 (46.8)	11 594 (51.6)	
Smoking						
Never	38 897 (50.4)	5063 (5.0)	12 030 (52.5)	10 989 (50.7)	10 815 (48.1)	<.001
Past	33 646 (43.6)	4050 (4.0)	9145 (39.9)	9662 (44.5)	10 789 (48.0)	
Current	4663 (6.0)	1014 (1.0)	1729 (7.6)	1054 (4.9)	866 (3.9)	
Falls in past year						
0	52 393 (67.9)	6784 (67.0)	15377 (67.1)	14 780 (68.1)	15 452 (68.8)	<.001
1	15 397 (20.0)	2029 (2.1)	4618 (20.2)	4400 (20.3)	4350 (19.4)	
2	6271 (8.1)	814 (8.0)	1952 (8.5)	1751 (8.1)	1754 (7.8)	
≥3	3145 (4.1)	500 (4.9)	957 (4.2)	774 (3.6)	914 (4.1)	
Alcohol use and frequency						
Never	8046 (10.4)	1458 (14.4)	2793 (12.2)	2176 (10.0)	1619 (7.2)	<.001
Past	13 923 (18.0)	2457 (24.2)	4620 (20.2)	3510 (16.2)	3336 (14.8)	
<1 Drink/mo	9010 (11.7)	1529 (15.1)	2872 (12.5)	2525 (11.6)	2084 (9.3)	
1 Drink/mo to <1 drink/wk	15 564 (20.2)	1938 (19.1)	4651 (20.3)	4449 (20.5)	4526 (20.1)	
1 Drink/wk to <7 drinks/wk	20 474 (26.5)	1747 (17.3)	5467 (23.9)	6061 (27.9)	7199 (32.1)	
≥7 Drinks/wk	10 189 (13.2)	998 (9.8)	2501 (10.9)	2984 (13.7)	3706 (16.5)	
Weight, kg						
≤58.9	15 621 (20.2)	1388 (13.7)	3869 (16.9)	4538 (20.9)	5826 (25.9)	<.001
59.0-65.2	15 704 (20.3)	1479 (14.6)	4020 (17.5)	4643 (21.4)	5562 (24.8)	
65.3-72.2	15 749 (20.4)	1776 (17.5)	4509 (19.7)	4650 (21.4)	4814 (21.4)	
72.3-82.5	15 419 (20.0)	2146 (21.2)	5051 (22.1)	4365 (20.1)	3857 (17.2)	
>82.5	14 713 (19.1)	3338 (33.0)	5455 (23.8)	3509 (16.2)	2411 (10.7)	
Height, cm						
≤156.4	14 834 (19.2)	2151 (21.2)	4624 (20.2)	3997 (18.4)	4065 (18.1)	<.001
156.5-160.0	15 293 (19.8)	1951 (19.3)	4646 (20.3)	4292 (19.8)	4409 (19.6)	
160.1-163.4	15 822 (20.5)	2116 (20.9)	4615 (20.1)	4488 (20.7)	4604 (20.5)	
163.5-167.0	15 502 (20.1)	1996 (19.7)	4464 (19.5)	4455 (20.5)	4600 (20.5)	
>167.0	15 755 (20.4)	1913 (18.9)	4555 (19.9)	4483 (20.7)	4807 (21.4)	
History of diabetes						
No	73 191 (94.8)	9337 (92.2)	21 415 (93.5)	20 511 (95.4)	21 728 (96.7)	<.001
Yes	4015 (5.2)	790 (7.8)	1489 (6.5)	998 (4.6)	742 (3.3)	
Age at menopause, mean (SD), y	48.2 (6.4)	47.5 (6.7)	47.9 (6.5)	48.4 (6.2)	48.7 (6.1)	<.001
History of fracture after age 55 y						
No	68 455 (88.7)	9009 (89.0)	20 281 (88.5)	19 250 (88.7)	19 915 (88.6)	.72
Yes	8751 (11.3)	1118 (11.0)	2623 (11.5)	2455 (11.3)	2555 (11.4)	
History of any fracture						
No	48 322 (62.6)	6447 (63.6)	14 423 (63.0)	13 603 (62.7)	13 849 (61.6)	.001
Yes	28 884 (37.4)	3680 (36.4)	8481 (37.0)	8102 (37.3)	8621 (38.4)	
History of osteoporosis						
No	70 721 (91.6)	9236 (91.2)	20 934 (91.4)	19 860 (91.5)	20 650 (91.9)	.10
Yes	6485 (8.4)	891 (8.8)	1970 (8.6)	1845 (8.5)	1820 (8.1)	
Lifetime hormone therapy use, y						
0	28 980 (37.5)	4134 (40.8)	9164 (40.0)	7925 (36.5)	7757 (34.5)	<.001
0.1-5.0	19 518 (25.3)	2574 (25.4)	5603 (24.5)	5476 (25.3)	5865 (26.1)	
5.1-10.0	11 387 (14.7)	1307 (12.9)	3223 (14.1)	3280 (15.1)	3577 (15.9)	
>10.0	17 321 (22.4)	2112 (2.9)	4914 (21.4)	5024 (23.1)	5271 (23.5)	
Bone drug use						
No	74 220 (96.1)	9801 (96.8)	22 087 (96.4)	20 821 (95.9)	21 511 (95.7)	<.001
Yes	2986 (3.9)	326 (3.2)	817 (3.6)	884 (4.1)	959 (4.3)	
Corticosteroid use						
No	76 194 (98.7)	9907 (97.8)	22 533 (98.4)	21 451 (98.8)	22 303 (99.3)	<.001
Yes	1012 (1.3)	220 (2.2)	371 (1.6)	254 (1.2)	167 (0.7)	
Thiazide use						
No	73 114 (94.7)	9469 (93.5)	21 553 (94.1)	20 598 (94.9)	21 526 (95.8)	<.001
Yes	4092 (5.3)	658 (6.5)	1351 (5.9)	1107 (5.1)	944 (4.2)	
Calcium intake, mg						
≤618.3	14 815 (19.2)	2872 (28.3)	5165 (22.5)	3619 (16.7)	3178 (14.1)	<.001
618.4-930.7	15 330 (19.9)	2241 (22.1)	4962 (21.6)	4269 (19.6)	3878 (17.2)	
930.8-1276.9	15 604 (20.2)	1997 (19.7)	4624 (20.2)	4512 (20.8)	4486 (20.0)	
1277.0-1751.5	15 630 (20.2)	1604 (15.8)	4286 (18.7)	4636 (21.3)	5112 (22.7)	
>1751.5	15 827 (20.5)	1429 (14.1)	3896 (17.0)	4696 (21.6)	5831 (25.9)	
Vitamin D intake, IU						
≤121.0	15 014 (19.4)	2571 (25.4)	4903 (21.4)	3765 (17.3)	3775 (16.8)	<.001
121.1-234.3	15 307 (19.8)	2233 (22.1)	4848 (21.2)	4206 (19.4)	4020 (17.9)	
234.4-471.0	15 410 (20.0)	1933 (19.1)	4518 (19.7)	4419 (20.4)	4540 (20.2)	
471.1-609.4	15 634 (20.2)	1757 (17.4)	4473 (19.5)	4582 (21.1)	4822 (21.5)	
>609.4	15 841 (20.5)	1633 (16.1)	4162 (18.2)	4733 (21.8)	5313 (23.6)	
Physical function score >90						
No	47 069 (61.0)	7785 (76.9)	16 237 (70.9)	12 994 (59.9)	10 053 (44.7)	<.001
Yes	30 137 (39.0)	2342 (23.1)	6667 (29.1)	8711 (40.1)	12 417 (55.3)	

Abbreviation: MET, metabolic equivalent.

^a^ Based on *t* tests for continuous variables and χ^2^ tests for categorical variables.

During a mean (SD) follow-up period of 14.0 (5.2) years, 25 516 women (33.1%) reported experiencing at least 1 fracture. Table 2 presents site-specific fracture event rates and HRs according to categories of total PA. Compared with inactive women (ie, 0 MET h/wk) and adjusted for covariates and sedentary time, the HRs for total fracture were 0.94 (95% CI, 0.90-0.98) for women with more than 0 to 7.5 MET h/wk, 0.95 (95% CI, 0.91-0.99) for women with more than 7.5 to 17.7 MET h/wk, and 0.94 (95% CI, 0.90-0.98) for women with more than 17.7 MET h/wk (*P *for trend = .16). Women in the highest total PA tertile had an 18% lower risk of hip fracture (HR, 0.82; 95% CI, 0.72-0.95; *P* for trend < .001). Knee fracture was positively associated with total PA (highest tertile vs inactive: HR, 1.26; 95% CI, 1.05-1.50; *P *for trend = .08). Elbow fracture was positively associated with PA (highest tertile vs inactive: HR, 1.11; 95% CI, 0.91-1.35; *P *for trend = .02). Sensitivity analysis limiting follow-up time to the first 10 years showed a stronger inverse association for total PA with hip fracture (highest tertile vs inactive: HR, 0.62; 95% CI, 0.51-0.77; *P *for trend < .001) and an inverse association with total fracture risk (HR, 0.93; 95% CI, 0.88-0.99; *P *for trend = .40); however, the associations with knee (HR, 1.09; 95% CI, 0.85-1.40; *P *for trend = .78) and elbow fracture (HR, 1.12; 95% CI, 0.86-1.47; *P *for trend = .05) were no longer evident (eTable 1 in the Supplement).

**Table 2.** Associations of Total Recreational Physical Activity With Total and Site-Specific Fractures

Model	Adjusted HR (95% CI)				*P* Value^a^
	0 MET h/wk (n = 10 127)	>0 to 7.5 MET h/wk (n = 22 904)	>7.5 to 17.7 MET h/wk (n = 21 705)	>17.7 MET h/wk (n = 22 470)	
MET h/wk, median (range)	0 (0-0)	3.8 (0.5-7.5)	12.5 (7.6-17.7)	27.3 (17.8-142.3)	NA
**Total Fracture: 25 355 Events**					
Events, No. (annualized %)	3164 (2.86)	7278 (2.79)	7315 (2.82)	7598 (2.75)	NA
Age	1 [Reference]	0.94 (0.91-0.98)	0.94 (0.90-0.98)	0.92 (0.88-0.96)	<.001
Multivariable^b^	1 [Reference]	0.94 (0.90-0.98)	0.95 (0.91-0.99)	0.94 (0.90-0.98)	.16
**Hip Fracture: 2673 Events**					
Events, No. (annualized %)	320 (0.24)	847 (0.28)	784 (0.26)	722 (0.22)	NA
Age	1 [Reference]	1.03 (0.90-1.17)	0.93 (0.82-1.06)	0.81 (0.71-0.93)	<.001
Multivariable^b^	1 [Reference]	1.01 (0.89-1.15)	0.92 (0.80-1.05)	0.82 (0.72-0.95)	<.001
**Wrist or Forearm Fracture: 5473 Events**					
Events, No. (annualized %)	643 (0.50)	1481 (0.49)	1595 (0.53)	1754 (0.55)	NA
Age	1 [Reference]	0.96 (0.87-1.05)	1.03 (0.94-1.13)	1.07 (0.97-1.17)	.005
Multivariable^b^	1 [Reference]	0.93 (0.85-1.02)	0.98 (0.89-1.08)	1.00 (0.92-1.11)	.11
**Clinical Vertebral Fracture: 4056 Events**					
Events, No. (annualized %)	504 (0.39)	1201 (0.39)	1165 (0.38)	1186 (0.37)	NA
Age	1 [Reference]	0.95 (0.86-1.06)	0.90 (0.81-1.00)	0.86 (0.78-0.96)	.002
Multivariable^b^	1 [Reference]	0.96 (0.86-1.07)	0.92 (0.83-1.02)	0.91 (0.82-1.02)	.10
**Elbow Fracture: 1207 Events**					
Events, No. (annualized %)	146 (0.11)	311 (0.10)	337 (0.11)	413 (0.13)	NA
Age	1 [Reference]	0.89 (0.73-1.09)	0.96 (0.79-1.16)	1.10 (0.91-1.32)	.02
Multivariable^b^	1 [Reference]	0.90 (0.74-1.09)	0.96 (0.79-1.18)	1.11 (0.91-1.35)	.02
**Foot Fracture: 3859 Events**					
Events, No. (annualized %)	503 (0.39)	1094 (0.36)	1104 (0.37)	1158 (0.36)	NA
Age	1 [Reference]	0.93 (0.83-1.03)	0.93 (0.84-1.03)	0.91 (0.82-1.01)	.18
Multivariable^b^	1 [Reference]	0.93 (0.84-1.03)	0.94 (0.84-1.05)	0.94 (0.84-1.05)	.66
**Hand Fracture: 947 Events**					
Events, No. (annualized %)	111 (0.08)	295 (0.10)	246 (0.08)	295 (0.09)	NA
Age	1 [Reference]	1.10 (0.89-1.37)	0.90 (0.72-1.13)	1.01 (0.81-1.25)	.51
Multivariable^b^	1 [Reference]	1.11 (0.89-1.39)	0.93 (0.74-1.17)	1.06 (0.84-1.34)	.97
**Knee Fracture: 1664 Events**					
Events, No. (annualized %)	179 (0.14)	506 (0.16)	452 (0.15)	527 (0.16)	NA
Age	1 [Reference]	1.17 (0.99-1.39)	1.02 (0.86-1.22)	1.12 (0.94-1.32)	.83
Multivariable^b^	1 [Reference]	1.20 (1.01-1.43)	1.11 (0.93-1.32)	1.26 (1.05-1.50)	.08
**Lower Leg Fracture: 4140 Events**					
Events, No. (annualized %)	559 (0.44)	1252 (0.42)	1134 (0.38)	1195 (0.37)	NA
Age	1 [Reference]	0.95 (0.86-1.05)	0.86 (0.77-0.95)	0.84 (0.76-0.93)	<.001
Multivariable^b^	1 [Reference]	0.97 (0.88-1.07)	0.90 (0.81-1.00)	0.92 (0.82-1.02)	.10
**Pelvis Fracture: 1664 Events**					
Events, No. (annualized %)	182 (0.14)	449 (0.15)	501 (0.16)	532 (0.16)	NA
Age	1 [Reference]	0.97 (0.82-1.16)	1.06 (0.90-1.26)	1.06 (0.90-1.26)	.19
Multivariable^b^	1 [Reference]	0.90 (0.76-1.08)	0.93 (0.78-1.11)	0.91 (0.76-1.09)	.66
**Tailbone Fracture: 546 Events**					
Events, No. (annualized %)	74 (0.06)	166 (0.05)	162 (0.05)	144 (0.04)	NA
Age	1 [Reference]	0.90 (0.69-1.19)	0.86 (0.65-1.13)	0.71 (0.54-0.94)	.009
Multivariable^b^	1 [Reference]	0.96 (0.73-1.27)	0.97 (0.73-1.29)	0.87 (0.65-1.18)	.03
**Upper Arm Fracture: 2964 Events**					
Events, No. (annualized %)	373 (0.29)	877 (0.29)	823 (0.27)	891 (0.27)	NA
Age	1 [Reference]	0.95 (0.84-1.08)	0.88 (0.78-0.99)	0.89 (0.79-1.01)	.06
Multivariable^b^	1 [Reference]	1.00 (0.88-1.13)	0.97 (0.86-1.10)	1.03 (0.91-1.18)	.45
**Upper Leg Fracture: 1147 Events**					
Events, No. (annualized %)	136 (0.10)	354 (0.11)	321 (0.10)	336 (0.10)	NA
Age	1 [Reference]	1.02 (0.84-1.25)	0.90 (0.73-1.09)	0.88 (0.72-1.07)	.05
Multivariable^b^	1 [Reference]	1.01 (0.83-1.23)	0.88 (0.72-1.08)	0.88 (0.71-1.08)	.08
**Other Fracture: 8288 Events**					
Events, No. (annualized %)	1019 (0.80)	2304 (0.77)	2428 (0.82)	2537 (0.80)	NA
Age	1 [Reference]	0.93 (0.86-1.00)	0.95 (0.88-1.02)	0.92 (0.86-0.99)	.19
Multivariable^b^	1 [Reference]	0.92 (0.86-1.00)	0.96 (0.89-1.03)	0.95 (0.88-1.02)	.88

Abbreviations: HR, hazard ratio; MET, metabolic equivalent; NA, not applicable.

^a^ Derived from a separate survival model with the outcome of interest as a function of linear trend across group medians.

^b^ Adjusted for age, race/ethnicity, education, smoking status, alcohol use, height, weight, history of fracture after age 55 years, bone drug use, corticosteroid use, calcium intake, vitamin D intake, lifetime hormone therapy use (years), falls in the past year, physical function construct, thiazide use, diabetes, age at menopause, history of osteoporosis, and sedentary time.

Associations between walking amount and a subset of major osteoporotic fractures appear in eTable 2 in the Supplement. Adjusting for covariates, other types of PA, and sedentary time, hip fracture was inversely associated with walking categories (>0 to 3.5 MET h/wk: HR, 0.99; 95% CI, 0.89-1.11; >3.5 to 7.5 MET h/wk: HR, 0.92; 95% CI, 0.83-1.02; >7.5 MET h/wk: HR, 0.88; 95% CI, 0.78-0.98; *P* for trend = .01). There was no association between walking amount and clinical vertebral fracture. Walking was not clearly associated with total or wrist and forearm fracture.

Risk of fracture according to PA intensity appears in eTable 3 and eTable 4 in the Supplement. Following adjustment for covariates, moderate, strenuous, and walking activities and sedentary time, inverse associations were observed for mild activity with hip fracture (HR, 0.82; 95% CI, 0.73-0.93; *P* for trend = .003), clinical vertebral fracture (HR, 0.87; 95% CI, 0.78-0.96; *P* for trend = .006), and total fracture (HR, 0.91; 95% CI, 0.87-0.94; *P *for trend < .001) among women with more than 3.5 MET h/wk compared with women with no mild PA (eTable 3 in the Supplement). When combining moderate and strenuous activity and walking 2 mph or faster into moderate to vigorous PA (MVPA), women with MVPA comparable with guideline recommendations (ie, ≥9 MET h/wk) had lower hip fracture risk (HR, 0.88; 95% CI, 0.81-0.96; *P *for trend = .002) but higher risk of wrist or forearm fracture (HR, 1.09; 95% CI: 1.03-1.15; *P *for trend = .004) than women with less than 9 MET h/wk MVPA (eTable 4 in the Supplement). Moderate to vigorous physical activity was not associated with risks of total fracture or clinical vertebral fracture.

We next examined nonrecreational activity, including yard work and heavy household chores. In models adjusted for total recreational PA and sedentary time, more than 6 MET h/wk of yard work was associated with lower risk of total fracture (HR, 0.95; 95% CI, 0.92-0.98; *P *for trend = .002) and hip fracture (HR, 0.90; 95% CI, 0.82-0.99; *P *for trend = .04) compared with no yard work (Table 3). Yard work was not associated with risks of clinical vertebral or wrist and forearm fractures. Energy expenditure from heavy chores was not associated with total or site-specific fractures (eTable 5 in the Supplement).

**Table 3.** Associations of Yard Work With Hip, Wrist or Forearm, Clinical Vertebral, and Total Fractures

Model	Adjusted HR (95% CI)			*P* Value^a^
	0 MET h/wk (n = 38 538)	>0 to 6 MET h/wk (n = 21 301)	>6 MET h/wk (n = 17 367)	
MET h/wk, median (range)	0 (0-0)	3.3 (1.3-5.3)	13.3 (7.3-44.0)	NA
**Total Fracture: 25 355 Events**				
Events, No. (annualized %)	12 436 (2.83)	7037 (2.74)	5882 (2.79)	NA
Age	1 [Reference]	0.97 (0.94-1.00)	0.96 (0.93-0.99)	.03
Multivariable^b^	1 [Reference]	0.97 (0.94-1.00)	0.95 (0.92-0.98)	.002
**Hip Fracture: 2673 Events**				
Events, No. (annualized %)	1348 (0.26)	720 (0.24)	605 (0.24)	NA
Age	1 [Reference]	0.96 (0.88-1.05)	0.91 (0.82-1.00)	.05
Multivariable^b^	1 [Reference]	0.96 (0.87-1.05)	0.90 (0.82-0.99)	.04
**Wrist or Forearm Fracture: 5473 Events**				
Events, No. (annualized %)	2582 (0.51)	1546 (0.52)	1345 (0.55)	NA
Age	1 [Reference]	1.04 (0.97-1.10)	1.08 (1.01-1.15)	.03
Multivariable^b^	1 [Reference]	1.02 (0.96-1.08)	1.03 (0.96-1.10)	.47
**Clinical Vertebral Fracture: 4056 Events**				
Events, No. (annualized %)	2020 (0.39)	1074 (0.36)	962 (0.39)	NA
Age	1 [Reference]	0.92 (0.85-0.99)	0.96 (0.89-1.04)	.38
Multivariable^b^	1 [Reference]	0.94 (0.87-1.01)	0.97 (0.89-1.05)	.49

Abbreviations: HR, hazard ratio; MET, metabolic equivalent; NA, not applicable.

^a^ Derived from a separate survival model with the outcome of interest as a function of linear trend across group medians.

^b^ Adjusted for age, race/ethnicity, education, smoking status, alcohol use, height, weight, history of fracture after age 55 years, bone drug use, corticosteroid use, calcium intake, vitamin D intake, lifetime hormone therapy use (years), falls in the past year, physical function construct, thiazide use, diabetes, age at menopause, history of osteoporosis, recreational physical activity, and sedentary time.

Table 4 presents associations between sedentary behavior and fracture risks. In age-adjusted models, 9.5 hours of daily sitting or lying down was associated with higher risks of hip fracture (HR, 1.11; 95% CI, 1.01-1.21; *P *for trend = .03), clinical vertebral fracture (HR, 1.09; 95% CI, 1.01-1.17; *P *for trend = .02), wrist or forearm fracture (HR, 1.07; 95% CI, 1.01-1.14; *P *for trend = .11), and total fracture (HR, 1.10; 95% CI, 1.07-1.13; *P *for trend < .001). Associations were attenuated and not statistically significant in the multivariable-adjusted models, with the exception of total fracture risk, which was attenuated but remained statistically significant, including when further adjusted for total PA (HR, 1.04; 95% CI, 1.01-1.07; *P *for trend = .01).

**Table 4.** Associations of Sedentary Behavior With Hip, Wrist or Forearm, Clinical Vertebral, and Total Fractures

Model	Adjusted HR (95% CI)			*P* Value^a^
	<6.5 h/d (n = 25 506)	≥6.5 to 9.5 h/d (n = 22 523)	>9.5 h/d (n = 29 177)	
Sedentary h/d, median (range)	5.0 (0-6.5)	8.0 (7.0-9.5)	12.0 (10.0-24.0)	NA
**Total Fracture: 25 355 Events**				
Events, No. (annualized %)	8002 (2.71)	7478 (2.82)	9875 (2.85)	NA
Age	1 [Reference]	1.04 (1.00-1.07)	1.10 (1.07-1.13)	<.001
Multivariable^b^	1 [Reference]	1.00 (0.97-1.03)	1.04 (1.01-1.07)	.01
**Hip Fracture: 2673 Events**				
Events, No. (annualized %)	866 (0.25)	828 (0.26)	979 (0.24)	NA
Age	1 [Reference]	1.04 (0.95-1.14)	1.11 (1.01-1.21)	.03
Multivariable^b^	1 [Reference]	0.98 (0.89-1.08)	1.02 (0.93-1.12)	.57
**Wrist or Forearm Fracture: 5473 Events**				
Events, No. (annualized %)	1753 (0.52)	1575 (0.52)	2145 (0.53)	NA
Age	1 [Reference]	0.99 (0.92-1.06)	1.07 (1.01-1.14)	.02
Multivariable^b^	1 [Reference]	0.97 (0.90-1.03)	1.05 (0.98-1.12)	.11
**Clinical Vertebral Fracture: 4056 Events**				
Events, No. (annualized %)	1297 (0.38)	1214 (0.39)	1545 (0.38)	NA
Age	1 [Reference]	1.02 (0.95-1.11)	1.09 (1.01-1.17)	.02
Multivariable^b^	1 [Reference]	0.98 (0.90-1.06)	1.02 (0.94-1.10)	.60

Abbreviations: HR, hazard ratio; NA, not applicable.

^a^ Derived from a separate survival model with the outcome of interest as a function of linear trend across group medians.

^b^ Adjusted for age, race/ethnicity, education, smoking status, alcohol use, height, weight, history of fracture after age 55 years, bone drug use, corticosteroid use, calcium intake, vitamin D intake, lifetime hormone therapy use (years), falls in the past year, physical function construct, thiazide use, diabetes, age at menopause, history of osteoporosis, and total recreational physical activity.

We next evaluated risk of total fracture according to jointly classified total PA and sedentary time exposures (Figure). Fracture was inversely associated with total PA, regardless of time spent sedentary.

**Figure.**
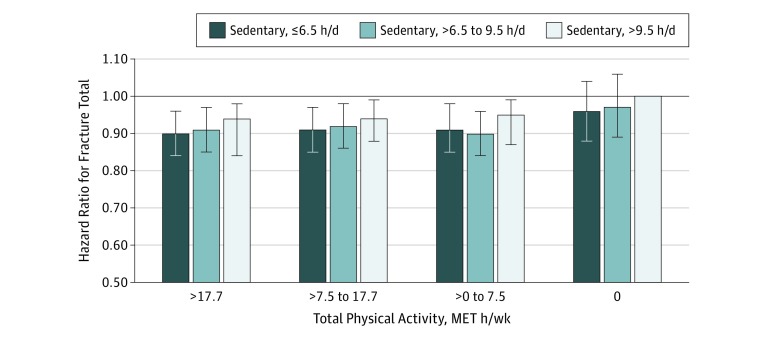
Risk of Total Fracture According to Jointly Classified Sedentary Behavior and Total Physical Activity Exposures Models are adjusted for age, race/ethnicity, education, smoking status, alcohol use, height, weight, history of fracture after age 55 years, bone drug use, corticosteroid use, calcium intake, vitamin D intake, lifetime hormone therapy use, falls in the past year, physical function construct, thiazide use, diabetes, age at menopause, and history of osteoporosis. The reference category was sedentary for more than 9.5 h/d and 0 metabolic equivalent (MET) h/wk physical activity. Whiskers represent 95% CIs.

We examined whether the associations of total PA with hip, wrist or forearm, clinical vertebral, and total fracture differed after stratifying on categories of age, race/ethnicity, and fall frequency history at baseline. There were no significant interactions observed (data not shown).

Finally, to evaluate the robustness of results from our primary analysis that used baseline PA and sedentary exposures, we repeated the analysis using time-varying exposures based on updated information collected after baseline (eTable 6 and eTable 7 in the Supplement). Adjusting for covariates and sedentary time, total PA was significantly inversely associated with total fracture (>0 to 7.5 MET hr/wk: HR, 0.97; 95% CI, 0.93-1.005; >7.5 to 17.7 MET h/wk: HR, 0.96; 95% CI, 0.92-1.003; >17.7 MET h/wk: HR, 0.94; 95% CI, 0.90-0.98; *P *for trend = .007), hip fracture (>0 to 7.5 MET hr/wk: HR, 0.95; 95% CI, 0.84-1.06; >7.5 to 17.7 MET h/wk: HR, 0.80; 95% CI, 0.71-0.90; >17.7 MET h/wk: HR, 0.69; 95% CI, 0.61-0.79; *P *for trend < .001), clinical vertebral fracture (>0 to 7.5 MET hr/wk: HR, 0.97; 95% CI, 0.88-1.07; >7.5 to 17.7 MET h/wk: HR, 0.99; 95% CI, 0.89-1.09; >17.7 MET h/wk: HR, 0.88; 95% CI, 0.80-0.98; *P *for trend = .01), and upper leg fracture (>0 to 7.5 MET hr/wk: HR, 0.73; 95% CI, 0.61-0.88; >7.5 to 17.7 MET h/wk: HR, 0.85; 95% CI, 0.71-1.02; >17.7 MET h/wk: HR, 0.71; 95% CI, 0.59-0.86; *P *for trend = .03). Total PA was positively associated with wrist or forearm fracture (>0 to 7.5 MET hr/wk: HR, 0.97; 95% CI, 0.88-1.06; >7.5 to 17.7 MET h/wk: HR, 1.05; 95% CI, 0.96-1.15; >17.7 MET h/wk: HR, 1.09; 95% CI, 0.99-1.19; *P *for trend = .003) (eTable 6 in the Supplement). Controlling for covariates and total PA, time-varying sedentary time was positively associated only with total fracture (≥6.5 to 9.5 h/d: HR, 1.00; 95% CI, 0.97-1.03; >9.5 h/d: HR, 1.06; 95% CI, 1.03-1.10; *P *for trend < .001) (eTable 7 in the Supplement).

## Discussion

This large cohort study among older, community-dwelling, ambulatory women found that recreational and nonrecreational PA was inversely associated with risks of hip, clinical vertebral, and total fractures. Total PA was positively associated with knee and elbow fracture. Mild-intensity PA was associated with lower risks of hip, vertebral, and total fracture, and MVPA was associated with lower risk of hip fracture but higher risk of wrist or forearm fracture. Yard work was inversely associated with hip and total fractures. Results of time-varying exposure analysis were materially the same for sedentary time but were somewhat stronger for PA compared with those for baseline exposures. Taken with results of sensitivity analyses showing somewhat stronger results for PA when restricting follow-up to the initial 10 years, it could be that PA in the recent term is most relevant to fracture. To our knowledge, this is the most comprehensive evaluation of PA and fracture incidence in older women.

Of the more than 50 available studies on PA and fracture, relatively few are prospective studies in older women that have assessed various PA types and fracture risks at multiple body sites.^17,18,19,20,21,22,23,24,25,26,27^ Our study supports the consistent evidence of an inverse association of PA with hip fracture. We extended understanding by demonstrating associations of PA with fractures at other sites previously studied infrequently or not at all.

Vertebral fracture has poor long-term morbidity and mortality^28^; thus, its prevention is imperative. Moderate to vigorous PA has been inversely associated with radiographic vertebral fractures in older women.^20^ In a 2-year randomized exercise trial,^29^ incidence of vertebral fracture was higher in the control group than in the intervention group. Conversely, 2 other studies^30,31^ did not find an association between PA and vertebral fracture. Our study suggests that risk of vertebral fracture was not increased with greater amount or intensity of PA and that mild intensity PA is associated with reduced risk of clinical vertebral fractures in later life. Additional research is needed on a potential role for PA, even at lighter intensities, in the prevention of this fracture type that carries poor prognosis in older adults.

The inverse association of PA with hip and clinical vertebral fractures is biologically plausible. Physical activity could attenuate the age-related reduction in spine and hip BMD.^6,32,33,34^ Regular PA can help improve balance, range of motion, and muscle strength,^35^ thereby reducing falls,^36^ a major risk factor for fracture.^37^ Because hip and vertebral fractures occur frequently in older women, even a modest protective association with PA could account for a meaningful number of averted fracture cases and related complications within the population. While ambulatory PA can mitigate age-related hip and spine bone loss, it may exert less stress on the wrist and forearm and minimally influence BMD at this site. The greater demands of MVPA might be assumed to increase the risk of falling. However, recent results in a WHI substudy^38^ on women aged 63 to 99 years whose PA was directly measured using accelerometers showed that fall rates were elevated in women engaging in low but not moderate or higher levels of MVPA. Women capable of doing MVPA may be more functional and more likely to break a fall with outstretched hands, which could account for the higher prevalence of wrist and forearm fractures associated with MVPA in the present study.

The 2018 revised PA guidelines^39^ recommend regular participation in MVPA to maintain and promote health in adults. It has been suggested that even mild (ie, light) activity could be beneficial for older adults.^40^ Our study found that mild PA and walking were associated with lower risk of hip fracture in older women. This is an important and relatively novel finding. To date, there has been insufficient evidence available to support recommending lighter intensity activities as part of public health guidelines.^6,7,40^ If other studies confirm our results showing that light-intensity activity is associated with fracture benefit, there could be basis for a future guideline recommendation. Mild PA and walking account for the majority of daily activity time in WHI participants.^41^ Lower-intensity activities are more easily adopted by older individuals and should be recommended when such activity is not contraindicated.

A positive association between sedentary behavior and total fracture risk was observed in the present study, even after controlling for fracture risk factors and total recreational PA. Women reporting more than 9.5 h/d of sedentary time experienced a 4% higher risk of all fractures combined compared with women with the least amount of sedentary time. When jointly classified with total PA, fracture risk associated with sedentary behavior was no longer present. To our knowledge, few studies have been published on the association of sedentary time with fracture in adults.^42,43,44,45^ Results have been mixed in men. A 2014 study^42^ reported a 38% lower multivariable (including total PA) adjusted relative risk of hip fracture; another^45^ reported a more than 2-fold higher risk of hip fracture and a 68% higher risk of total fracture when comparing inactive men with their most active counterparts. Results in women are also inconsistent, with 1 study^43^ showing no association between weekly sitting time and hip fracture in younger postmenopausal women and another^44^ showing a 35% higher multivariable relative risk of hip fracture in women who are sedentary compared with highly active women, aged 20 to 93 years. Prolonged time spent in sedentary behaviors is associated with reduced physical functioning^12^ and leg blood flow,^46^ which could predispose individuals to falls,^47^ reduced bone quality,^19^ and fracture.^48^ On the other hand, lack of movement because of more time spent sedentary would theoretically reduce the opportunity for a fracture event while standing or during ambulation. Inconsistency of study results may partly reflect the difficulty of assessing sedentary behavior using questionnaires, particularly in older adults and women. A recent accelerometer substudy in WHI suggested time spent sedentary is considerably more than what is self-reported in women aged 63 to 99 years.^49^ Evaluation of fracture risks associated with sedentary behavior measured objectively is needed.

### Strengths and Limitations

Strengths of our study include its prospective design, large sample size, and long follow-up period with low loss to follow-up. We were able to distinguish between various types and intensities of PA; sedentary behavior was also assessed. Mutual adjustment for PA and sedentary behavior when analyzing each exposure provides an approach for teasing out these interrelated factors of fracture incidence. Repeated assessments of PA and sedentary time permitted time-varying exposure analysis, the results of which were similar to those for baseline sedentary exposure and somewhat stronger than those for baseline PA.

Limitations include the use of self-report questionnaires to assess PA; misclassification on exposure is inevitable.^49^ Because the PA (and sedentary behavior) assessment preceded fracture occurrence, exposure misclassification might be expected to be nondifferential and any resulting bias of associations toward the null,^50^ but this may not be the case in all such circumstance.^51^ The questionnaire reliability,^10,13^ similarity of results for baseline and time-varying exposure analyses, and previously observed associations with major disease end points affecting older women^12,52,53^ enhances confidence in the study results. Fracture outcome ascertainment was based only on self-report after 2010 in the WHI. High validity for self-reported hip (78%) and wrist or forearm (81%) fractures, but lower validity (51%) for vertebral fractures has been reported in WHI.^15^ Physical activity can be influenced by multilevel sociocultural and ecological forces,^54^ which are challenging to quantify and account for statistically in studies such as ours. Further, we conducted many analyses, and some associations could be owing to chance alone; results should be interpreted accordingly.

## Conclusions

In this cohort study, greater amounts of total PA were associated with lower risk of total fracture, but associations varied by fracture site. Greater MVPA was associated with lower risk of hip fracture but higher risk of wrist or forearm fracture. Mild activity was inversely associated with risks of hip, clinical vertebral, and total fracture, independent of other PA and sedentary behavior. The current results suggest that lower-intensity activities, including walking and nonrecreational activities, could have benefit on fracture risk at older ages. If confirmed, future recommendations on fracture prevention in postmenopausal women should promote light PA, especially in those who are frail and unable to safely engage in more intense activities. Sedentary behavior as an independent factor predisposing individuals to fracture requires further investigation.

## Notes

Supplement. **eTable 1. **Associations Between Total Recreational Physical Activity and Incidence of Total and Site-Specific Fractures Limited to First 10 Years of Follow-up **eTable 2. **Associations Between Walking and Incidence of Hip, Wrist or Forearm, Clinical Vertebral, and Total Fractures **eTable 3. **Associations Between Mild Physical Activity and Incidence of Hip, Wrist or Forearm, Clinical Vertebral, and Total Fractures **eTable 4. **Associations Between Moderate to Vigorous Physical Activity and Incidence of Hip, Wrist or Forearm, Clinical Vertebral, and Total Fractures **eTable 5. **Associations Between Heavy Chores and Incidence of Hip, Wrist or Forearm, Clinical Vertebral, and Total Fractures **eTable 6. **Associations Between Time-Dependent Total Recreational Physical Activity and Total and Site-Specific Fractures **eTable 7. **Associations Between Time-Dependent Sedentary Behavior and Hip, Wrist or Forearm, Clinical Vertebral, and Total Fractures **eTable 8. **Tabulation of Exclusions and Missing Covariate Information
