# Supplementary material for: Association of Physical Activity and Fracture Risk Among Postmenopausal Women
Source: JAMA Netw Open. 2019 Oct 25;2(10):e1914084. doi: 10.1001/jamanetworkopen.2019.14084 (PMC6822158; doi:10.1001/jamanetworkopen.2019.14084)
Supplement: Supplement. — eTable 1. Associations Between Total Recreational Physical Activity and Incidence of Total and Site-Specific Fractures Limited to First 10 Years of Follow-up eTable 2. Associations Between Walking and Incidence of Hip, Wrist or Forearm, Clinical Vertebral, and Total Fractures eTable 3. Associations Between Mild Physical Activity and Incidence of Hip, Wrist or Forearm, Clinical Vertebral, and Total Fractures eTable 4. Associations Between Moderate to Vigorous Physical Activity and Incidence of Hip, Wrist or Forearm, Clinical Vertebral, and Total Fractures eTable 5. Associations Between Heavy Chores and Incidence of Hip, Wrist or Forearm, Clinical Vertebral, and Total Fractures eTable 6. Associations Between Time-Dependent Total Recreational Physical Activity and Total and Site-Specific Fractures eTable 7. Associations Between Time-Dependent Sedentary Behavior and Hip, Wrist or Forearm, Clinical Vertebral, and Total Fractures eTable 8. Tabulation of Exclusions and Missing Covariate Information [file jamanetwopen-2-e1914084-s001.pdf]

Supplementary Online Content

LaMonte MJ, Wactawski-Wende J, Larson JC, et al; Women's Health Initiative (WHI). Association of physical activity and fracture risk among postmenopausal women. *JAMA Netw Open*. 2019;2(10):e1914084. doi:10.1001/jamanetworkopen.2019.14084

eTable 1. Associations Between Total Recreational Physical Activity and Incidence of Total and Site-Specific Fractures Limited to First 10 Years of Follow-up

eTable 2. Associations Between Walking and Incidence of Hip, Wrist or Forearm, Clinical Vertebral, and Total Fractures

eTable 3. Associations Between Mild Physical Activity and Incidence of Hip, Wrist or Forearm, Clinical Vertebral, and Total Fractures

eTable 4. Associations Between Moderate to Vigorous Physical Activity and Incidence of Hip, Wrist or Forearm, Clinical Vertebral, and Total Fractures

eTable 5. Associations Between Heavy Chores and Incidence of Hip, Wrist or Forearm, Clinical Vertebral, and Total Fractures

eTable 6. Associations Between Time-Dependent Total Recreational Physical Activity and Total and Site-Specific Fractures

eTable 7. Associations Between Time-Dependent Sedentary Behavior and Hip, Wrist or Forearm, Clinical Vertebral, and Total Fractures

eTable 8. Tabulation of Exclusions and Missing Covariate Information

This supplementary material has been provided by the authors to give readers additional information about their work.

© 2019 LaMonte MJ et al. *JAMA Network Open.*

eTable 1. Associations Between Total Recreational Physical Activity and Incidence of Total and Site-Specific Fractures Limited to First 10 Years of Follow-up

Fracture Outcome	Total Physical Activity (MET-hr/wk)				*P-value*[a]
	0	>0 - 7.5	7.6 - 17.7	> 17.7	
N	10,127	22,904	21,705	22,470	
Median [range] (MET-hr/wk)	0 [0, 0]	3.8 [0.5, 7.5]	12.5 [7.6, 17.7]	27.33 [17.7, 142.3]	
**Total Fracture (14,590 cases)**					
Events (Ann %)	1973 (2.04)	4284 (1.90)	4098 (1.85)	4235 (1.81)	
Age Adjusted HR (95% CI)	1.00	0.92 (0.87, 0.97)	0.91 (0.86, 0.96)	0.90 (0.85, 0.95)	0.008
Multivariable adjusted HR (95% CI)	1.00	0.92 (0.88, 0.98)	0.92 (0.87, 0.97)	0.93 (0.88, 0.99)	0.40
**Hip Fracture (1,119 cases)**					
Events (Ann %)	164 (0.15)	384 (0.15)	330 (0.13)	241 (0.09)	
Age Adjusted HR (95% CI)	1.00	0.94 (0.79, 1.13)	0.85 (0.70, 1.02)	0.62 (0.50, 0.75)	<0.001
Multivariable adjusted HR (95% CI)	1.00	0.93 (0.78, 1.12)	0.84 (0.70, 1.02)	0.62 (0.51, 0.77)	<0.001
**Wrist / Forearm Fracture (3,152 cases)**					
Events (Ann %)	390 (0.37)	842 (0.34)	902 (0.37)	1018 (0.40)	
Age Adjusted HR (95% CI)	1.00	0.92 (0.82, 1.04)	1.02 (0.90, 1.15)	1.11 (0.99, 1.25)	<0.001
Multivariable adjusted HR (95% CI)	1.00	0.90 (0.80, 1.02)	0.99 (0.87, 1.11)	1.07 (0.95, 1.21)	0.002
**Clinical Vertebral Fracture (1,795 cases)**					
Events (Ann %)	248 (0.23)	537 (0.21)	511 (0.21)	499 (0.19)	
Age Adjusted HR (95% CI)	1.00	0.89 (0.77, 1.04)	0.87 (0.75, 1.02)	0.83 (0.71, 0.97)	0.04
Multivariable adjusted HR (95% CI)	1.00	0.90 (0.78, 1.05)	0.90 (0.77, 1.06)	0.90 (0.76, 1.05)	0.46
**Elbow Fracture (634 cases)**					
Events (Ann %)	83 (0.08)	168 (0.07)	176 (0.07)	207 (0.08)	
Age Adjusted HR (95% CI)	1.00	0.87 (0.67, 1.13)	0.94 (0.72, 1.22)	1.06 (0.82, 1.36)	0.13
Multivariable adjusted HR (95% CI)	1.00	0.88 (0.68, 1.15)	0.97 (0.74, 1.27)	1.12 (0.86, 1.47)	0.05
**Foot Fracture (2,267 cases)**					
Events (Ann %)	320 (0.30)	654 (0.26)	648 (0.27)	645 (0.25)	
Age Adjusted HR (95% CI)	1.00	0.89 (0.78, 1.02)	0.91 (0.80, 1.04)	0.86 (0.75, 0.98)	0.10
Multivariable adjusted HR (95% CI)	1.00	0.89 (0.78, 1.02)	0.92 (0.80, 1.05)	0.88 (0.77, 1.02)	0.31
**Hand Fracture (427 cases)**					
Events (Ann %)	47 (0.04)	141 (0.06)	103 (0.04)	136 (0.05)	
Age Adjusted HR (95% CI)	1.00	1.28 (0.92, 1.78)	0.96 (0.68, 1.35)	1.22 (0.87, 1.70)	0.71
Multivariable adjusted HR (95% CI)	1.00	1.30 (0.93, 1.81)	0.99 (0.70, 1.41)	1.30 (0.92, 1.85)	0.43
**Knee Fracture (795 cases)**					
Events (Ann %)	97 (0.09)	264 (0.10)	208 (0.08)	226 (0.09)	
Age Adjusted HR (95% CI)	1.00	1.16 (0.92, 1.46)	0.93 (0.73, 1.19)	0.98 (0.77, 1.24)	0.18
Multivariable adjusted HR (95% CI)	1.00	1.19 (0.94, 1.50)	1.01 (0.79, 1.29)	1.09 (0.85, 1.40)	0.78
**Lower Leg Fracture (2,565 cases)**					
Events (Ann %)	368 (0.35)	776 (0.31)	701 (0.29)	720 (0.28)	
Age Adjusted HR (95% CI)	1.00	0.92 (0.81, 1.04)	0.85 (0.75, 0.96)	0.83 (0.73, 0.94)	0.006
Multivariable adjusted HR (95% CI)	1.00	0.94 (0.83, 1.07)	0.91 (0.80, 1.03)	0.91 (0.80, 1.05)	0.31
**Pelvis Fracture (680 cases)**					
Events (Ann %)	87 (0.08)	204 (0.08)	198 (0.08)	191 (0.07)	
Age Adjusted HR (95% CI)	1.00	0.97 (0.75, 1.24)	0.96 (0.75, 1.24)	0.91 (0.71, 1.17)	0.43
Multivariable adjusted HR (95% CI)	1.00	0.90 (0.70, 1.16)	0.86 (0.66, 1.11)	0.80 (0.62, 1.05)	0.13
**Tailbone Fracture (215 cases)**					
Events (Ann %)	28 (0.03)	70 (0.03)	64 (0.03)	53 (0.02)	

© 2019 LaMonte MJ et al. *JAMA Network Open.*

Age Adjusted HR (95% CI)	1.00	1.06 (0.68, 1.64)	0.99 (0.64, 1.54)	0.79 (0.50, 1.25)	0.12
Multivariable adjusted HR (95% CI)	1.00	1.13 (0.73, 1.76)	1.11 (0.71, 1.76)	0.94 (0.58, 1.53)	0.46
**Upper Arm Fracture (1,382 cases)**					
Events (Ann %)	203 (0.19)	421 (0.17)	382 (0.16)	376 (0.14)	
Age Adjusted HR (95% CI)	1.00	0.87 (0.74, 1.03)	0.81 (0.69, 0.96)	0.78 (0.65, 0.92)	0.01
Multivariable adjusted HR (95% CI)	1.00	0.90 (0.76, 1.07)	0.88 (0.74, 1.05)	0.88 (0.73, 1.05)	0.32
**Upper Leg Fracture (386 cases)**					
Events (Ann %)	65 (0.06)	118 (0.05)	116 (0.05)	87 (0.03)	
Age Adjusted HR (95% CI)	1.00	0.75 (0.55, 1.01)	0.75 (0.56, 1.02)	0.55 (0.40, 0.76)	0.001
Multivariable adjusted HR (95% CI)	1.00	0.73 (0.54, 0.99)	0.73 (0.54, 1.01)	0.55 (0.39, 0.78)	0.004
**Other Fracture (3,135 cases)**					
Events (Ann %)	413 (0.39)	913 (0.37)	878 (0.36)	931 (0.36)	
Age Adjusted HR (95% CI)	1.00	0.94 (0.84, 1.05)	0.92 (0.82, 1.04)	0.94 (0.84, 1.05)	0.56
Multivariable adjusted HR (95% CI)	1.00	0.95 (0.85, 1.07)	0.95 (0.84, 1.07)	1.00 (0.88, 1.13)	0.52

[a]p-value derived from a separate survival model with the outcome of interest as a function of linear trend across group medians.

Multivariable Model is adjusted for age, race/ethnicity, education, smoking status, alcohol use, height, weight, history of fracture after 55 years old, bone drug use, corticosteroid use, calcium intake, vitamin D intake, lifetime hormone therapy use, falls in past year, physical function construct, thiazide use, diabetes, age at menopause, history of osteoporosis, and sedentary time.

© 2019 LaMonte MJ et al. *JAMA Network Open.*

eTable 2. Associations Between Walking and Incidence of Hip, Wrist or Forearm, Clinical Vertebral, and Total Fractures

Fracture Outcome	Walking Amount (MET-hr/wk)												P-value[a]
	0			0+ - 3.5			3.5+ - 7.5			> 7.5			
N	22,798			14,974			21,029			18,405			
Median [range] (MET-hr/wk)	0 [0, 0]			1.7 [0.5, 3.5]			5.0 [3.6, 7.5]			12.5 [8.3, 40.8]			
**Total Fracture (25,355 cases)**													
Events (Ann %)	7260 (2.79)			4927 (2.86)			6943 (2.78)			6225 (2.76)			
Age Adjusted HR (95% CI)	1.00			1.00	(0.96,	1.04)	0.96	(0.93,	1.00)	0.97	(0.93,	1.00)	0.02
Multivariable adjusted HR (95% CI)	1.00			1.00	(0.96,	1.03)	0.97	(0.93,	1.00)	0.99	(0.96,	1.03)	0.46
**Hip Fracture (2,673 cases)**													
Events (Ann %)	774 (0.25)			541 (0.27)			761 (0.26)			597 (0.22)			
Age Adjusted HR (95% CI)	1.00			0.98	(0.88,	1.10)	0.93	(0.84,	1.03)	0.86	(0.77,	0.96)	0.003
Multivariable adjusted HR (95% CI)	1.00			0.99	(0.89,	1.11)	0.92	(0.83,	1.02)	0.88	(0.78,	0.98)	0.01
**Wrist / Forearm Fracture (5,473 cases)**													
Events (Ann %)	1489 (0.50)			1016 (0.51)			1522 (0.53)			1446 (0.56)			
Age Adjusted HR (95% CI)	1.00			1.01	(0.93,	1.09)	1.04	(0.97,	1.12)	1.11	(1.03,	1.19)	0.003
Multivariable adjusted HR (95% CI)	1.00			0.99	(0.91,	1.07)	1.00	(0.93,	1.08)	1.06	(0.98,	1.14)	0.11
**Clinical Vertebral Fracture (4,056 cases)**													
Events (Ann %)	1148 (0.38)			829 (0.41)			1138 (0.39)			941 (0.36)			
Age Adjusted HR (95% CI)	1.00			1.04	(0.95,	1.14)	0.97	(0.89,	1.05)	0.91	(0.83,	0.99)	0.007
Multivariable adjusted HR (95% CI)	1.00			1.05	(0.96,	1.15)	0.99	(0.91,	1.07)	0.96	(0.87,	1.05)	0.17

HR, hazard ratio; CI: confidence interval

[a]p-value derived from a separate survival model with the outcome of interest as a function of linear trend across group medians.

Multivariable model is adjusted for age, race/ethnicity, education, smoking status, alcohol use, height, weight, history of fracture after 55 years old, bone drug use, corticosteroid use, calcium intake, vitamin D intake, lifetime hormone therapy use, falls in past year, physical function construct, thiazide use, diabetes, age at menopause, history of osteoporosis, mild physical activity, moderate physical activity, strenuous physical activity, and sedentary time.

© 2019 LaMonte MJ et al. *JAMA Network Open.*

eTable 3. Associations Between Mild Physical Activity and Incidence of Hip, Wrist or Forearm, Clinical Vertebral, and Total Fractures

Fracture Outcome	Mild Physical Activity (MET-hr/wk)									*P-value*[a]
	0			>0 - 3.5			> 3.5			
N	55,434			13,234			8,538			
Median [range] (MET-hr/wk)	0 [0, 0]			2.5 [0.5, 3.5]			7.0 [4.5, 21.0]			
**Total Fracture (25,355 cases)**										
Events (Ann %)	18143	(2.81)		4448	(2.80)		2764	(2.69)		
Age Adjusted HR (95% CI)	1.00			1.01	(0.97,	1.04)	0.92	(0.88,	0.96)	<0.001
Multivariable adjusted HR (95% CI)	1.00			0.99	(0.96,	1.03)	0.91	(0.87,	0.94)	<0.001
**Hip Fracture (2,673 cases)**										
Events (Ann %)	1934	(0.25)		451	(0.24)		288	(0.24)		
Age Adjusted HR (95% CI)	1.00			0.99	(0.89,	1.10)	0.84	(0.74,	0.95)	0.007
Multivariable adjusted HR (95% CI)	1.00			0.98	(0.88,	1.08)	0.82	(0.73,	0.93)	0.003
**Wrist / Forearm Fracture (5,473 cases)**										
Events (Ann %)	3903	(0.52)		962	(0.52)		608	(0.52)		
Age Adjusted HR (95% CI)	1.00			1.01	(0.94,	1.09)	0.96	(0.88,	1.04)	0.38
Multivariable adjusted HR (95% CI)	1.00			1.00	(0.93,	1.07)	0.93	(0.86,	1.02)	0.14
**Clinical Vertebral Fracture (4,056 cases)**										
Events (Ann %)	2930	(0.39)		687	(0.37)		439	(0.37)		
Age Adjusted HR (95% CI)	1.00			0.97	(0.89,	1.05)	0.88	(0.79,	0.97)	0.01
Multivariable adjusted HR (95% CI)	1.00			0.96	(0.88,	1.04)	0.87	(0.78,	0.96)	0.006

HR, hazard ratio; CI: confidence interval.

[a]p-value derived from a separate survival model with the outcome of interest as a function of linear trend across group medians.

[b]Model 1: adjusted for age, race/ethnicity, education, smoking status, alcohol use, height, weight, history of fracture after 55 years old, bone drug use, corticosteroid use, calcium intake, vitamin D intake, lifetime hormone therapy use, falls in past year, physical function construct, thiazide use, diabetes, age at menopause, history of osteoporosis, moderate physical activity, strenuous physical activity, walking, and sedentary time.

© 2019 LaMonte MJ et al. *JAMA Network Open.*

eTable 4. Associations Between Moderate-to-Vigorous Physical Activity and Incidence of Hip, Wrist or Forearm, Clinical Vertebral, and Total Fractures

Fracture Outcome	Moderate-to-Vigorous Physical Activity (MET-hr/wk)						*P-value*[a]
	< 9			≥ 9			
N	39929			37277			
Median [range] (MET-hr/wk)	1.9 [0, 8.9]			18.3 [9.0, 121.3]			
**Total Fracture (25355 cases)**							
Events (Ann %)	12617	(2.78)		12738	(2.81)		
Age Adjusted HR (95% CI)	1.00			0.99	(0.97,	1.01)	0.38
Multivariate adjusted HR (95% CI)	1.00			1.01	(0.99,	1.04)	0.38
**Hip Fracture (2,673 cases)**							
Events (Ann %)	1408	(0.26)		1265	(0.23)		
Age Adjusted HR (95% CI)	1.00			0.87	(0.81,	0.94)	<0.001
Multivariate adjusted HR (95% CI)	1.00			0.88	(0.81,	0.96)	0.002
**Wrist / Forearm Fracture (5,473 cases)**							
Events (Ann %)	2568	(0.49)		2905	(0.56)		
Age Adjusted HR (95% CI)	1.00			1.12	(1.06,	1.18)	<0.001
Multivariate adjusted HR (95% CI)	1.00			1.09	(1.03,	1.15)	0.004
**Clinical Vertebral Fracture (4,056 cases)**							
Events (Ann %)	2054	(0.39)		2002	(0.38)		
Age Adjusted HR (95% CI)	1.00			0.94	(0.88,	1.00)	0.04
Multivariate adjusted HR (95% CI)	1.00			0.97	(0.91,	1.04)	0.40

HR, hazard ratio; CI: confidence interval

[a]p-value derived from a separate survival model with the outcome of interest as a function of linear trend across group medians.

Multivariable model is adjusted for age, race/ethnicity, education, smoking status, alcohol use, height, weight, history of fracture after 55 years old, bone drug use, corticosteroid use, calcium intake, vitamin D intake, lifetime hormone therapy use, falls in past year, physical function construct, thiazide use, diabetes, age at menopause, history of osteoporosis, and sedentary time.

© 2019 LaMonte MJ et al. *JAMA Network Open.*

eTable 5. Associations Between Heavy Chores and Incidence of Hip, Wrist or Forearm, Clinical Vertebral, and Total Fractures

**Fracture Outcome**	**Heavy Household Chores (MET-hr/wk)**									***P-value*[a]**
	0			>0 - 7			> 7			
N	26309			34537			16360			
Median [range] (MET-hr/wk)	0 [0, 0]			7 [6.6, 7]			17.5 [17.5, 42.0]			
**Total Fracture (25,355 cases)**										
Events (Ann %)	8839	(2.85%)		11219	(2.75%)		5297	(2.81%)		
Age Adjusted HR (95% CI)	1.00			0.95	(0.93,	0.98)	0.96	(0.93,	1.00)	0.02
Multivariate adjusted HR (95% CI)	1.00			0.98	(0.95,	1.01)	0.99	(0.96,	1.03)	0.73
**Hip Fracture (2,673 cases)**										
Events (Ann %)	941	(0.26%)		1114	(0.23%)		618	(0.28%)		
Age Adjusted HR (95% CI)	1.00			0.87	(0.80,	0.95)	1.01	(0.91,	1.12)	0.87
Multivariate adjusted HR (95% CI)	1.00			0.89	(0.82,	0.97)	1.04	(0.94,	1.15)	0.46
**Wrist / Forearm Fracture (5,473 cases)**										
Events (Ann %)	1833	(0.51%)		2487	(0.53%)		1153	(0.53%)		
Age Adjusted HR (95% CI)	1.00			1.03	(0.97,	1.09)	1.02	(0.95,	1.10)	0.62
Multivariate adjusted[e] HR (95% CI)	1.00			1.05	(0.98,	1.11)	1.03	(0.96,	1.12)	0.38
**Clinical Vertebral Fracture (4,056 cases)**										
Events (Ann %)	1362	(0.37%)		1822	(0.38%)		872	(0.40%)		
Age Adjusted HR (95% CI)	1.00			1.00	(0.93,	1.07)	1.01	(0.93,	1.10)	0.82
Multivariate adjusted[e] HR (95% CI)	1.00			1.04	(0.96,	1.11)	1.04	(0.95,	1.14)	0.35

HR, hazard ratio; CI: confidence interval.

[a]p-value derived from a separate survival model with the outcome of interest as a function of linear trend across group medians.

Multivariable model is adjusted for age, race/ethnicity, education, smoking status, alcohol use, height, weight, history of fracture after 55 years old, bone drug use, corticosteroid use, calcium intake, vitamin D intake, lifetime hormone therapy use, falls in past year, physical function construct, thiazide use, diabetes, age at menopause, history of osteoporosis, total recreational physical activity, and sedentary time.

© 2019 LaMonte MJ et al. *JAMA Network Open.*

eTable 6. Associations Between Time-Dependent Total Recreational Physical Activity and Total and Site-Specific Fractures

	Total Physical Activity (MET-hr/wk)				P-Value[a]
	**0**	**>0 - 7.5**	**>7.5 - 17.7**	**> 17.7**	
Median [range] (MET-hr/wk)	0 [0, 0]	3.8 [0.5, 7.5]	12.5 [7.6, 17.7]	27.3 [17.8, 142.3]	
**Total Fracture (25,355 cases)**					
Events (Ann %)	3638 (2.95)	7427 (2.83)	7065 (2.81)	7225 (2.68)	
Age adjusted HR (95% CI)	1.00	0.96 (0.92, 1.00)	0.95 (0.91, 0.99)	0.91 (0.87, 0.95)	<0.001
Multivariable adjusted HR (95% CI)	1.00	0.97 (0.93, 1.00)	0.96 (0.92, 1.00)	0.94 (0.90, 0.98)	0.007
**Hip Fracture (2,673 cases)**					
Events (Ann %)	451 (0.31)	897 (0.29)	714 (0.24)	611 (0.19)	
Age adjusted HR (95% CI)	1.00	0.95 (0.85, 1.07)	0.80 (0.71, 0.90)	0.69 (0.61, 0.78)	<0.001
Multivariable adjusted HR (95% CI)	1.00	0.95 (0.84, 1.06)	0.80 (0.71, 0.90)	0.69 (0.61, 0.79)	<0.001
**Wrist/Forearm Fracture (5,473 cases)**					
Events (Ann %)	719 (0.50)	1488 (0.49)	1557 (0.54)	1709 (0.55)	
Age adjusted HR (95% CI)	1.00	0.99 (0.90, 1.08)	1.08 (0.99, 1.18)	1.13 (1.03, 1.23)	<0.001
Multivariable adjusted HR (95% CI)	1.00	0.97 (0.88, 1.06)	1.05 (0.96, 1.15)	1.09 (0.99, 1.19)	0.003
**Clinical Vertebral Fracture (4056 cases)**					
Events (Ann %)	616 (0.42)	1213 (0.40)	1162 (0.40)	1065 (0.34)	
Age adjusted HR (95% CI)	1.00	0.94 (0.86, 1.04)	0.94 (0.85, 1.04)	0.83 (0.75, 0.91)	<0.001
Multivariable adjusted HR (95% CI)	1.00	0.97 (0.88, 1.07)	0.99 (0.89, 1.09)	0.88 (0.80, 0.98)	0.01
**Elbow Fracture (1,207 cases)**					
Events (Ann %)	160 (0.11)	336 (0.11)	327 (0.11)	384 (0.12)	

© 2019 LaMonte MJ et al. *JAMA Network Open.*

eTable 6. Associations Between Time-Dependent Total Recreational Physical Activity and Total and Site-Specific Fractures (continued)

	Total Recreational Physical Activity (MET-hr/wk)				P-Value[a]
	**0**	**>0 - 7.5**	**>7.5 - 17.7**	**> 17.7**	
Age adjusted HR (95% CI)	1.00	1.01 (0.83, 1.21)	1.01 (0.84, 1.22)	1.11 (0.92, 1.33)	0.14
Multivariable adjusted HR (95% CI)	1.00	1.01 (0.84, 1.23)	1.03 (0.85, 1.25)	1.12 (0.92, 1.36)	0.14
**Foot Fracture (3,859 cases)**					
Events (Ann %)	523 (0.36)	1154 (0.38)	1043 (0.36)	1139 (0.36)	
Age adjusted HR (95% CI)	1.00	1.06 (0.95, 1.17)	0.98 (0.89, 1.09)	0.99 (0.89, 1.09)	0.24
Multivariable adjusted HR (95% CI)	1.00	1.07 (0.97, 1.19)	1.01 (0.91, 1.13)	1.04 (0.93, 1.16)	0.90
**Hand Fracture (947 cases)**					
Events (Ann %)	120 (0.08)	280 (0.09)	268 (0.09)	279 (0.09)	
Age adjusted HR (95% CI)	1.00	1.12 (0.90, 1.38)	1.10 (0.89, 1.36)	1.07 (0.86, 1.32)	0.97
Multivariable adjusted HR (95% CI)	1.00	1.15 (0.93, 0.43)	1.17 (0.94, 1.46)	1.16 (0.93, 1.46)	0.45
**Knee Fracture (1,664 cases)**					
Events (Ann %)	208 (0.14)	515 (0.17)	452 (0.15)	489 (0.15)	
Age adjusted HR (95% CI)	1.00	1.18 (1.01, 1.39)	1.07 (0.91, 1.26)	1.09 (0.93, 1.28)	0.77
Multivariable adjusted HR (95% CI)	1.00	1.22 (1.04, 1.43)	1.17 (0.99, 1.38)	1.23 (1.03, 1.45)	0.20
**Lower Leg Fracture (4,140 cases)**					
Events (Ann %)	620 (0.43)	1266 (0.42)	1103 (0.38)	1151 (0.37)	
Age adjusted HR (95% CI)	1.00	0.98 (0.89, 1.07)	0.88 (0.80, 0.97)	0.85 (0.77, 0.94)	<0.001
Multivariable adjusted HR (95% CI)	1.00	1.01 (0.92, 1.11)	0.94 (0.85, 1.04)	0.93 (0.84, 1.03)	0.05

© 2019 LaMonte MJ et al. *JAMA Network Open.*

eTable 6. Associations Between Time-Dependent Total Recreational Physical Activity and Total and Site-Specific Fractures (continued)

	Total Recreational Physical Activity (MET-hr/wk)				P-Value[a]
	0	>0 - 7.5	>7.5 - 17.7	> 17.7	
**Pelvis Fracture (1,664 cases)**					
Events (Ann %)	230 (0.16)	457 (0.15)	500 (0.17)	477 (0.15)	
Age adjusted HR (95% CI)	1.00	0.95 (0.81, 1.12)	1.10 (0.94, 1.29)	1.03 (0.88, 1.20)	0.37
Multivariable adjusted HR (95% CI)	1.00	0.89 (0.76, 1.05)	0.99 (0.84, 1.16)	0.90 (0.77, 1.07)	0.58
**Tailbone Fracture (546 cases)**					
Events (Ann %)	91 (0.06)	170 (0.05)	141 (0.05)	144 (0.04)	
Age adjusted HR (95% CI)	1.00	0.89 (0.69, 1.15)	0.76 (0.59, 0.99)	0.74 (0.57, 0.96)	0.02
Multivariable adjusted HR (95% CI)[c]	1.00	0.96 (0.74, 1.24)	0.89 (0.68, 1.17)	0.92 (0.70, 1.22)	0.61
**Upper Arm Fracture (2,964 cases)**					
Events (Ann %)	471 (0.32)	847 (0.27)	792 (0.27)	854 (0.27)	
Age adjusted HR (95% CI)	1.00	0.85 (0.76, 0.96)	0.83 (0.74, 0.93)	0.85 (0.76, 0.95)	0.11
Multivariable adjusted HR (95% CI)	1.00	0.91 (0.81, 1.02)	0.94 (0.83, 1.05)	1.00 (0.88, 1.12)	0.25
**Upper Leg Fracture (1,147 cases)**					
Events (Ann %)	199 (0.13)	307 (0.10)	346 (0.12)	295 (0.09)	
Age adjusted HR (95% CI)	1.00	0.73 (0.61, 0.88)	0.86 (0.72, 1.02)	0.71 (0.59, 0.85)	0.02
Multivariable adjusted HR (95% CI)	1.00	0.73 (0.61, 0.88)	0.85 (0.71, 1.02)	0.71 (0.59, 0.86)	0.03

© 2019 LaMonte MJ et al. *JAMA Network Open.*

eTable 6. Associations Between Time-Dependent Total Recreational Physical Activity and Total and Site-Specific Fractures (continued)

	**Total Recreational Physical Activity (MET-hr/wk)**				**P-Value[a]**
	**0**	**>0 - 7.5**	**>7.5 - 17.7**	**> 17.7**	
**Other Fracture (8288 cases)**					
Events (Ann %)	1211 (0.85)	2379 (0.79)	2303 (0.80)	2395 (0.78)	
Age adjusted HR (95% CI)	1.00	0.93 (0.87, 1.00)	0.92 (0.86, 0.98)	0.89 (0.83, 0.95)	0.003
Multivariable adjusted HR (95% CI)	1.00	0.94 (0.88, 1.01)	0.94 (0.87, 1.01)	0.92 (0.85, 0.99)	0.10

HR, hazard ratio; CI: confidence interval.
[a]p-value derived from a separate survival model with the outcome of interest as a function of linear trend across group medians.
Multivariable model is adjusted for age, race/ethnicity, education, smoking status, alcohol use, height, weight, history of fracture after 55 years old, bone drug use, corticosteroid use, calcium intake, vitamin D intake, lifetime hormone therapy use (years), falls in past year, physical function construct, thiazide use, diabetes, age at menopause, history of osteoporosis, and sedentary time.

© 2019 LaMonte MJ et al. *JAMA Network Open.*

eTable 7. Associations Between Time-Dependent Sedentary Behavior and Hip, Wrist or Forearm, Clinical Vertebral, and Total Fractures

	Sedentary Behavior (hour/day)			P-Value[a]
	**< 6.5**	**≥6.5 – 9.5**	**> 9.5**	
Median [range] (hours/day)	5.0 [0, 6.5]	8.0 [7.0, 9.5]	12.0 [10.0, 24.0]	
**Total Fracture (25,355 cases)**				
Events (Ann %)	9208 (2.73)	7688 (2.80)	8459 (2.86)	
Age adjusted HR (95% CI)	1.00	1.03 (1.00, 1.06)	1.13 (1.09, 1.16)	<0.001
Multivariable adjusted HR (95% CI)	1.00	1.00 (0.97, 1.03)	1.06 (1.03, 1.10)	<0.001
**Hip Fracture (n=2,673)**				
Events (Ann %)	1008 (0.25)	811 (0.25)	854 (0.24)	
Age adjusted HR (95% CI)	1.00	0.95 (0.87, 1.05)	1.09 (1.00, 1.20)	0.05
Multivariable adjusted HR (95% CI)	1.00	0.91 (0.83, 1.00)	1.02 (0.93, 1.12)	0.67
**Wrist/Forearm Fracture (n=5,473)**				
Events (Ann %)	1994 (0.52)	1699 (0.54)	1780 (0.52)	
Age adjusted HR (95% CI)	1.00	1.04 (0.97, 1.11)	1.07 (1.00, 1.14)	0.05
Multivariable adjusted HR (95% CI)	1.00	1.02 (0.96, 1.09)	1.05 (0.95, 1.11)	0.16
**Clinical Vertebral Fracture (n=4,056)**				
Events (Ann %)	1497 (0.38)	1248 (0.39)	1311 (0.38)	
Age adjusted HR (95% CI)	1.00	1.01 (0.94, 1.09)	1.11 (1.03, 1.20)	0.006
Multivariable adjusted HR (95% CI)	1.00	0.97 (0.90, 1.05)	1.03 (0.95, 1.11)	0.49

HR, hazard ratio; CI: confidence interval

[a]p-value derived from a separate survival model with the outcome of interest as a function of linear trend across group medians.

Multivariable model is adjusted for age, race/ethnicity, education, smoking status, alcohol use, height, weight, history of fracture after 55 years old, bone drug use, corticosteroid use, calcium intake, vitamin D intake, lifetime hormone therapy use (years), falls in past year, physical function construct, thiazide use, diabetes, age at menopause, history of osteoporosis, and, total recreational physical activity.

© 2019 LaMonte MJ et al. *JAMA Network Open.*

eTable 8. Tabulation of Exclusions and Missing Covariate Information

From the original 93 676 participants in the WHI Observational Study, 2674 were initially excluded due to a history of hip fracture, leaving 91 002

Of the 91 002, 3471 were excluded for missing physical activity / sedentary exposure data, leaving 87 531 participants.

Finally, 10 325 participants with missing covariate data were excluded for the following variables:

Variable	Missing
Height	683
Weight	427
Bone Drug Use	1
Smoking	951
Hormone Therapy Use	73
Fall History	457
Physical Function	1314
Education	684
Age at Menopause	3237
Calcium/Vitamin D Intake	3325
Alcohol Use	393

This results in the final sample of 77 206.

© 2019 LaMonte MJ et al. *JAMA Network Open.*
